# Transcatheter Tricuspid Valve Replacement: Current Options and Future Perspectives

**DOI:** 10.31083/RCM25712

**Published:** 2025-01-15

**Authors:** Guido Del Monaco, Gianluca Mincione, Alessandro Sticchi, Bernhard Reimers, Antonio Colombo, Antonio Mangieri

**Affiliations:** ^1^Cardiovascular Department IRCCS (Istituto di Ricerca e Cura a Carattere Scientifico) Humanitas Research Hospital, 20089 Rozzano, Milan, Italy; ^2^Department of Biomolecular Sciences, Humanitas University, 20072 Pieve Emanuele, Milan, Italy; ^3^Cardiac Catheterization Laboratory, Azienda Ospedaliero Universitaria Pisana, 56100 Pisa, Italy; ^4^Cardiothoracic and Vascular Department, University of Pisa, 56126 Pisa, Italy

**Keywords:** tricuspid valve, orthotopic tricuspid valve replacement, tricuspid regurgitation

## Abstract

Tricuspid regurgitation (TR) is a potentially lethal condition and represents a significant clinical challenge both for clinical and interventional cardiologists. Traditionally managed medically and surgically, transcatheter therapies are now an emerging option, especially in patients with prohibitive surgical risk due to age or comorbidities. Transcatheter tricuspid valve replacement (TTVR) is emerging as a potential solution for patients suffering from TR with positive clinical data supporting its use in a wide range of anatomies and clinical settings. However, the adoption of TTVR introduces new challenges, including a scarcity of long-term clinical risks of valve thrombosis, questions regarding the durability of implanted valves, and the potential higher risk for post-procedural pacemaker (PM) implantation.

## 1. Introduction

Tricuspid regurgitation (TR) is a significant heart valve disease, that affects around 4% of individuals aged 75 or older [1]. The clinical impact of TR is often underestimated due to its association with a poor outcome. However, TR embraces a broad spectrum of clinical and anatomical conditions with different prognoses. Historically, according to the etiology, TR has been categorized into primary or secondary. More recently, cardiac implantable electronic devices (CIED) related TR, promoted by leads that can hamper leaflet motion, has emerged as a relevant cause.

Primary TR, representing 10–15% of cases, results from structural abnormalities in tricuspid valve (TV) apparatus. This category encompasses various manifestations, including degenerative diseases such as leaflet flail or prolapse, congenital causes like Ebstein’s anomaly (detected in 8–10% of patients with severe TR [2, 3]), and rare acquired leaflet diseases resulting from conditions like carcinoid or rheumatic disease.

In contrast, secondary TR, representing a significant 80% of cases, is characterized by morphologically normal leaflets accompanied by annular dilatation and/or leaflet tethering. This category is further divided into ventricular secondary TR, often dependent on left heart disease (prevalent in 30–50% of patients with severe mitral regurgitation (MR) [4] and typically linked to secondary right heart failure (HF) [5]), and atrial secondary TR, associated with conditions such as atrial fibrillation (AF) or HF with preserved ejection fraction (HFpEF).

Numerous studies have reported a strong association between significant TR and an unfavorable prognosis [6, 7]. Despite surgery being the established treatment for TR, its efficacy in improving long-term survival over medical intervention may not always justify intervention, especially in high risk patients.

New percutaneous transcatheter approaches for TV repair and replacement have demonstrated promising clinical outcomes with acceptable rates of mortality and rehospitalization within the initial year of follow-up [8]. In particular, transcatheter tricuspid valve replacement (TTVR) represents a potential disruptive breakthrough allowing treatment of a broad spectrum of anatomies.

## 2. Indications for Transcatheter Tricuspid Valve Implantation

According to American and European guidelines, TV repair or replacement is recommended [9] alongside left-sided valve surgery in the following cases: (1) severe primary or secondary TR (class I); and (2) mild-to-moderate secondary TR with tricuspid annulus (TA) dilation (class IIa). As an isolated procedure, TV intervention is suggested for: (1) symptomatic severe primary TR; (2) asymptomatic severe primary TR with progressive right ventricular (RV) dysfunction (class IIb); and (3) symptomatic severe secondary TR in the absence of severe left ventricular (LV) or RV dysfunction and severe pulmonary arterial hypertension (class IIa).

The advent of transcatheter TV interventions (TTVI) in about the last ten years has offered an useful alternative to surgery in patients for whom surgery is unfeasible for high or prohibitive surgical risk. These TTVI methods are represented by: (1) direct or indirect TV restrictive annuloplasty; (2) transcatheter edge-to-edge repair (TEER, direct repair) or coaptation device (indirect restoration of leaflet coaptation); (3) heterotopic TV implantation; and (4) TTVR.

Currently the most commonly used percutaneous TV therapy, according to data coming from the registry, is TEER. Unfortunately, this approach is often unfeasible, mainly in patients with valvular anatomies with wide coaptation gaps, large annuli size or abnormal leaflet morphology. Consequently, TTVR can be a viable strategy in these scenarios, allowing it to address a wide range of anatomical variations.

### 2.1 Main Challenges in TTVR

TTVI shows specific issues for both the interventional cardiologist and imaging specialists. These challenges are first of all anatomical: the shape of TA is nonplanar and elliptical; moreover, the annulus is flexible and often severely dilated, and, compared with those of mitral valve (MV) regurgitant orifice areas are usually larger. The absence of annular calcification results in difficult anchoring and a lack of fluoroscopic landmarks. The TV is surrounded by critical structures that offer anatomical guidance but are susceptible to damage during intervention [10]. Another issue is represented by the closeness of the cardiac conduction system (atrioventricular node, bundle of His), which is located near the anteroseptal commissure in the membranous septum. In the same anatomical site as where the right coronary artery (RCA) where is located, is the TA which is useful as a radiological landmark. Specifically, the non-coronary sinus is located close to the anteroseptal commissure, while the postero-septal commissure is sited near to the coronary sinus ostium. Planning TTVI requires careful consideration of the TV’s anatomy, location, and characteristics. The TV forms with the inferior (IVC) and superior vena cava (SVC) a ~90° angle. This represents a significant challenge, as does the RV wall, which is thin and trabeculated and therefore prone to damage during intervention. The subvalvular apparatus, composed by several chordae and the moderator band, may create interference with device delivery. Furthermore, the presence of CIED necessitates thorough inclusion in disease assessment, device selection, and procedural risk stratification [11].

### 2.2 Patient Selection Criteria 

**Fig. 1.**
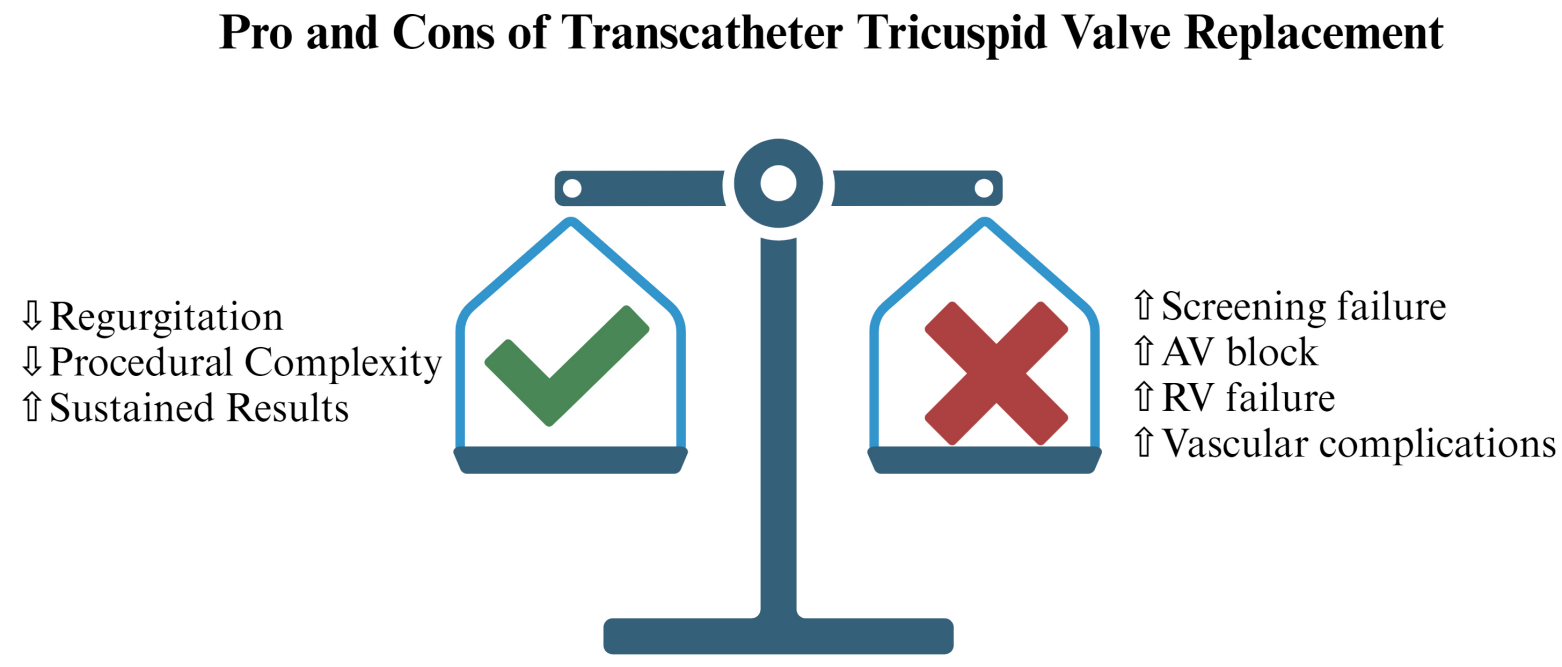
**Pro and Cons of Transcatheter Tricuspid Valve Replacement (TTVR)**. Legend: AV, atrio-ventricular; RV, right ventricular. Figure created by BioRender.

When considering whether repair or replacement is the better strategy, there are several factors to consider (Fig. 1). First of all, a coaptation gap >6–8 mm and eccentric regurgitant jets are associated with poor TEER procedural success and may suggest the selection of a TTVR strategy [12]. Moreover, an immobile or severely retracted leaflet are unlikely to have good outcomes with repair. TTVR may be more appropriate if residual TR is expected to be moderate or worse after repair. On the other hand, a complete abolition of TR, mainly in preload-dependent patients, may worsen a pre-existent RV failure due to afterload mismatch. CIED leads are not an absolute contraindication to TTVR but represent a common exclusion criterion for TEER, mainly due to an impingement with the tricuspid leaflet. To overcome this issue, CIED lead extraction may be a reasonable option, but currently, there are no prospective data about the impact of lead extraction on TR severity, and this strategy may result in worsening TR severity [13].

TV stenosis is an absolute contraindication for TEER because any repair strategy will increase the transvalvular gradient by reducing valve area. In these scenarios, TTVR could represent a valid alternative.

Other situations in which TEER is not a suitable option are congenital (such as Ebstein anomaly) or acquired conditions such as endocarditis, inflammatory diseases, or iatrogenic causes. The main issues of TTVR are first of all the presence of a very large or eccentric annulus, which may be prone to develop significant paravalvular leaks. RV and right atrium dimensions are crucial factors and should be large enough to facilitate the navigation of the device. Geometric parameters such as the height, position and angle between the IVC and the TA may contraindicate or make TTVR difficult. Moreover, in patients at high risk for bleeding, because lifelong anticoagulation is generally recommended after TTVR, a repair strategy may be the preferable strategy. Finally, a potential advantage of transcatheter valves is the potential to perform valve-in-valve TTVR at a future time but currently there is no long-term data on the durability of these devices [14].

## 3. Treatment Feasibility and Diagnostic Evaluation

### 3.1 Treatment Feasibility

Despite being a promising and effective therapy for severe TR management, TTVR has a high rate of screening failure and is not feasible in a high percentage of patients.

According to the TriACT registry, a real-world registry which compares all available options for patients with severe TR, almost 75% of patients are excluded from TTVR therapy mainly for cardiac computed tomography (CCT)-determined anatomical factors. The most frequent anatomical exclusion criteria is an enlarged TA (48%), followed by pacemaker (PM) lead impingement (9%), small right heart chamber dimensions (7%), an unfavorable IVC angle (3%) and flail leaflet (2%). Other reasons for screening failure include comorbidities (9%) and response to medical therapy (8%) [15].

### 3.2 Imaging in TTVI

Pre-procedural planning necessitates a comprehensive strategy, employing multi-modality imaging, which includes first of all transthoracic echocardiography (TTE) and transesophageal echocardiography (TEE), along with CCT imaging [16]. It is mandatory to evaluate disease severity under stable medical therapy, recognizing that TR severity is dependent on volume status but also on respiratory cycle and heart rate variations.

### 3.3 Echocardiography

According to guidelines, the etiology and severity of TR, and right chamber size and function should be assessed primarily by TTE and TEE (which should include transgastric and mid- and deep-esophageal views). Another imaging tool which is superior compared to two dimensional (2D) echocardiography is represented by three dimensional (3D) echocardiography, but is reliable only if performed by expert operators [17, 18].

Severe TR is defined by quantitative and semi-quantitative parameters: vena contracta (VC) width >0.7 cm, color jet area >10 cm^2^, proximal isovelocity surface area (PISA) radius >0.9 cm, effective regurgitant orifice area (EROA) >0.4 cm^2^, and regurgitant volume >45 mL.

A noteworthy adjustment in grading TR severity was made with the new proposed five grades scale, adding other two grades: massive (4+), and torrential (5+).

This updated classification system considers the extensive group of patients in recent studies whose echocardiographic measurements of severe TR far surpass the traditional criteria. These findings have been linked to negative RV remodeling and increased mortality [19, 20, 21].

### 3.4 Anatomic Considerations

#### Tricuspid Annulus Dilation

The choice of size and type of TTVR device requires an accurate sizing of TA, which is mainly made with 3D echocardiography or CCT scan. Semi-automated software allows a precise measurement of indirect planimetry, which has recently shown better agreement between TEE and CCT for TA sizing [22].

### 3.5 TV Leaflets Anatomy

The TV showcases anatomical variability, particularly in the anterior and posterior aspects. A simplified nomenclature, proposed by Hahn *et al*. [23], has recently emerged, offering valuable insights for pre-procedural planning and for the execution of transcatheter devices in TV interventions. The classification system outlines four major classes of leaflet morphologies: Type I represents the classic 3-leaflet morphology; Type II is the 2-leaflet morphology with fusion of the anterior and posterior leaflets; Type III is the 4-leaflet configuration with subcategories based on the location of the fourth leaflet; and Type IV involves more than four leaflets [23].

#### 3.5.1 Right Heart Morphology and Function 

RV enlargement is frequently observed in individuals with severe chronic TR [24]. A thorough evaluation of the RV should encompass measurements of its size and systolic performance. Traditional 2D echocardiography faces notable challenges in precisely assessing RV size and systolic function due to the RV’s complex structure and incomplete visualization in a single scanning plane, leading to variability. Consequently, several alternative indicators of systolic function have been introduced in 2D echocardiography, including: tricuspid annular plane systolic excursion (TAPSE), fractional area change (FAC), systolic velocity of the TA (S’) determined by Doppler tissue imaging, and, most recently, RV free-wall longitudinal strain [25].

#### 3.5.2 Ventriculo-Arterial Coupling 

RV contractility typically increases in response to rising afterload, maintaining RV-pulmonary artery (PA) coupling. However, when decompensation occurs, this adaptive mechanism fails, resulting in lower RV-PA coupling ratios, which are linked to poor outcomes. The gold standard for evaluating RV contractility and afterload includes end-systolic elastance (Ees) and arterial elastance (Ea). However, these parameters require invasive measurements with catheterization techniques that are not widely accessible [26]. Non-invasive parameters like TAPSE/pulmonary artery systolic pressure (PASP) have been validated as surrogates of invasive ones [27] and have shown promising results as prognostic markers in both medically and percutaneously treated TR patients. One study suggests that TAPSE/PASP are inversely correlated with one-year mortality in patients undergoing TTVR, indicating that higher baseline RV-PA coupling ratios might reflect greater tolerance to post-procedural afterload increases [28]. While echocardiography is a common method for estimating PASP, it has limitations, particularly in severe TR cases, where it can underestimate PASP due to large coaptation defects and early equalization of right chamber pressures. Right heart catheterization, which provides invasively measured PASP, offers a more accurate assessment, improving the prediction of post-procedural outcomes [29]. Thus, incorporating the invasive measure of PASP in RV-PA coupling assessments may refine patient selection for TTVR, providing better risk stratification and outcome predictions. Specifically, TAPSE/invasive PASP has been shown to predict survival independently of TR severity or baseline characteristics, offering a more reliable metric for guiding clinical decisions [30].

### 3.6 Other Interventional Considerations 

#### 3.6.1 Trans-Tricuspid CIED Leads

Approximately 25% of patients considered for TV intervention have a history of CIED implantation. Although this is not an absolute contraindication to the procedure, it is essential to verify that the leads have free and independent mobility and do not create an impingement with leaflet motion prior to the intervention. Lead extraction followed by the implantation of a leadless PM might be a preferable option to TV intervention in select patients experiencing lead-induced TR.

#### 3.6.2 Surrounding Structures

The main structures surrounding TV, which are also anatomic landmarks for interventional cardiologists, are represented by vena cava (both superior and inferior), the coronary sinus and RCA. Echocardiography (both TTE and TEE) is the first imaging tool for identifying these blood vessels but, due to its high spatial resolution and definition, CCT imaging may provide superior quality.

#### 3.6.3 Predictors of TR Recurrence

Imaging findings linked to adverse outcomes post-surgical TV repair may serve as valuable indicators for patient eligibility. While these parameters have been well studied and established for surgical TV repair [31] and for percutaneous TEER, evidence on TTVR is lacking. Insights from surgical predictors may guide considerations for similar outcomes in the percutaneous domain.

### 3.7 CCT

CCT has an indispensable role in evaluating annular size (useful for device selection), defining the landing zone of the device, assessing CIED lead course, determining RCA location [32], and examining sub-valvular structures. Additionally, it plays a pivotal role in determining appropriate access points, considering venous access site dimensions, tortuosity, IVC and SVC anatomy, and the approach to the TV [33]. Understanding the 3D course and angulation of the IVC is particularly crucial for delivery placement to achieve a coaxial approach, with the relationship between the IVC and TA being a significant determinant of technical success [33]. A maximal image quality is achieved with specific acquisition protocols but sometimes is challenging, mainly in patients with AF. Currently, to allow homogeneous opacification of right chambers and avoidance of artifacts, many protocols are available, based on parameters such as glomerular filtration rate, ejection fraction and weight. An indirect sign of severe TR which has been described as specific at CCT is early opacification of the IVC or hepatic veins. Direct quantification of TR is not feasible with CCT but several techniques have been proposed to estimate TR severity. These methods include calculation of regurgitant volume (analogously to cardiac magnetic resonance—CMR -, represented by the difference between RV and LV stroke volume), measurement of the anatomic regurgitant orifice area during mid-peak systole or calculation of the TA area at mid-diastole (cutoff >14 cm^2^ denotes severe TR). CCT has been compared to CMR in a study with a good correlation between the two techniques in assessing the right chamber volumes and ejection fraction [34]. For these reasons, CCT can be a valuable alternative to CMR, especially for patients with noncompatible intracardiac devices.

## 4. Medical Management of Tricuspid Regurgitation

Despite the importance of medical therapy in patients with severe TR, current guidelines advise that it should not delay interventions when indicated. Diuretic therapy, mainly represented by loop diuretics, is a mainstay for treating congestion in patients with relevant TR and is crucial to achieve an euvolemic status prior to considering any interventional treatment. In patients with TR and right heart failure (RHF) oral absorption of diuretics may be altered due to high central venous pressure, gastro-intestinal and renal congestion. Therefore, hospitalization for intravenous diuretic therapy may be required to achieve decongestion and to monitor the response with measurable parameters such as diuresis and natriuresis [35, 36]. In cases of loop diuretic resistance or inadequate response, combined diuretic therapy, along with inotropic agents and vasopressors, may be necessary, especially when peripheral hypoperfusion is present [35]. Although no specific neurohormonal modulators have demonstrated clear benefits in this clinical setting, a small observational study has suggested a potential association between sacubitril/valsartan and improved RV function [37]. Experimental data has also shown that mineralocorticoid receptor antagonists may help reduce RV afterload [38]. Additionally, a small randomized controlled trial in patients with heart failure and reduced ejection fraction (HFrEF) found that sodium–glucose cotransporter 2 inhibitors (SGLT2-i), in combination with other heart failure therapies, improved RV function more effectively than standard treatments alone [39]. It is crucial to remember that left-sided HFrEF may coexist with severe TR, therefore guideline-recommended medical therapy is a cornerstone in managing those patients.

## 5. Transcatheter Tricuspid Valve Replacement 

**Fig. 2.**
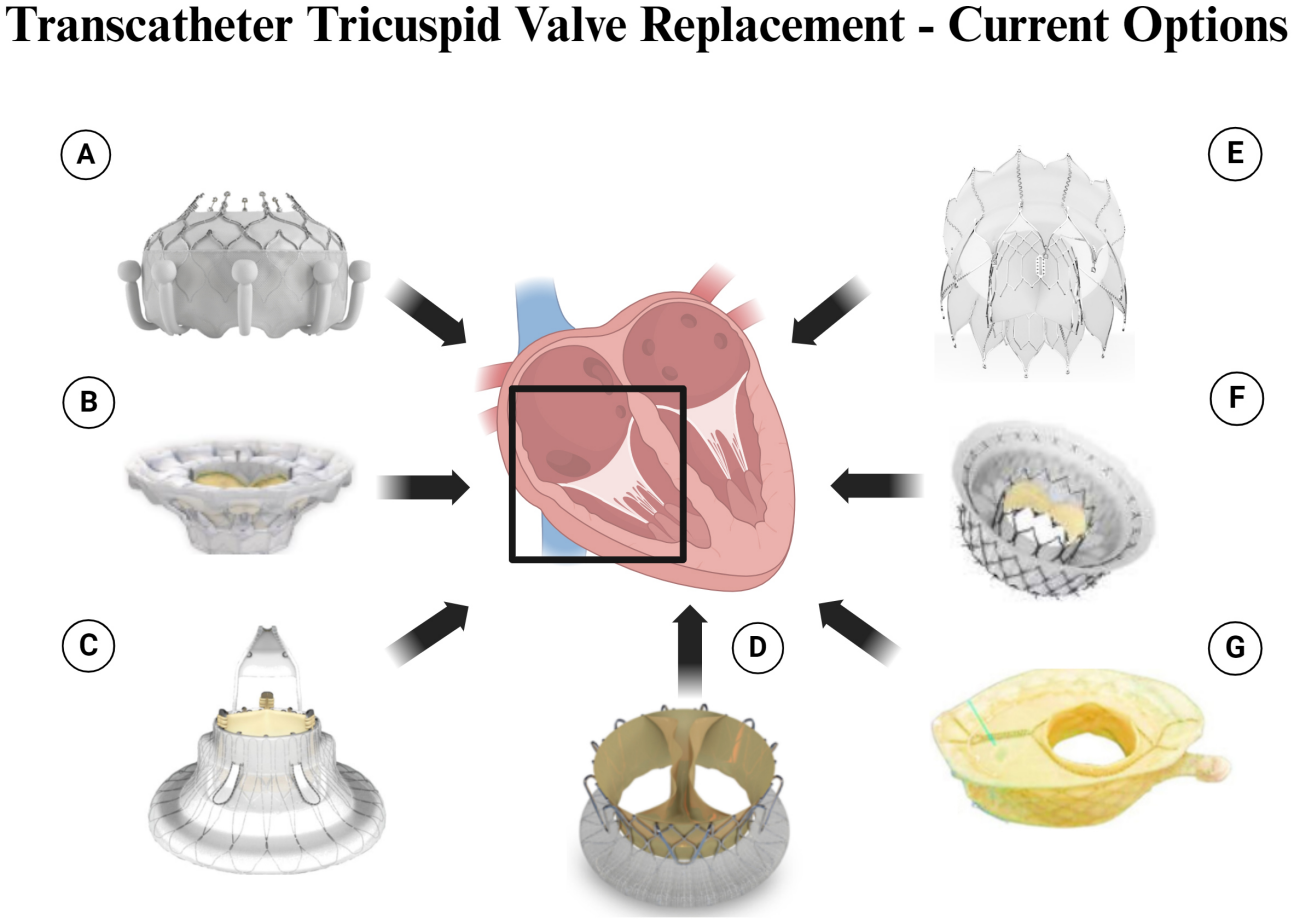
**Transcatheter Tricuspid Valve Replacement – Current Options**. Legend: (A) Evoque Valve. (B) Cardiovalve. (C) Lux Valve. (D) TriSol Valve. (E) Topaz Valve. (F) Intrepid Valve. (G) VDyne Valve. Figure created by BioRender.

Currently, various devices are under pre-clinical and clinical development for orthotopic TTVR (Table 1; Fig. 2).

**Table 1.** **Currently Available Transcatheter Tricuspid Replacement (TTVR) Devices and Respective Features**.

	Evoque	Cardiovalve	Lux Valve	TriSol	Topaz	Intrepid	Vdyne
Frame and Design	Nitinol frame with fabric skirt, nine anchors	Nitinol frame	Nitinol stent with atrial disc, interventricular septal anchor, two graspers	Cone-shaped nitinol frame with six fixation arms	Two stent frame crafted with nitinol	Dual-stent self-expanding nitinol	Nitinol frame
Anchoring	TV leaflets/Annulus	TV leaflet/atrial flange delivery	Septal anchor and anterior leaflet grasp	Tricuspid annulus	TV leaflets	Perimeter oversizing	TV Annulus, proximal loop and RVOT tab
Sizes (mm)	44, 48, 52	45, 50, 55, 60	50, 60, 70 (annulus)	62.5 (outflow)	<45 mm (annulus)	43, 46, 50	30 mm inner size with varying outer dimension (5 sizes 140 to 180 mm)
				50.3 (inflow)			
Access	Femoral	Femoral	Atrial/Mini-Thoracotomy/Jugular	Jugular	Femoral	Femoral/Apical	Femoral
Delivery system size (Fr)	28	28	32	30	29	35	29
Pericardial Leaflets	Bovine	Bovine	Bovine	Bovine	Bovine	Bovine	Porcine

Legend: TV, tricuspid valve; RVOT, right ventricular outflow tract.

### 5.1 Evoque

The EVOQUE system (Edwards Lifesciences) is constituted by a transfemoral venous system boasting a 28 Fr diameter, allowing the implantation of a bioprosthetic valve, which is available in three sizes (diameter 44 mm, 48 mm, or 52 mm). The delivery system is versatile and flexible and allows for the adjustment of depth. The bioprosthesis has a trileaflet design with a nitinol frame housing nine anchors and a fabric skirt and is crafted from bovine pericardium. The implantation procedure involves a pre-shaped guidewire which is moved towards the RV apex, ensuring its central alignment across the TV. This strategic placement lays the foundation for the subsequent deployment of the EVOQUE valve. The delivery system is then advanced to the right atrium (RA) and is flexed across the TV. Upon release, the position and trajectory are optimized advancing the delivery capsule beneath the valve, and anchors are exposed below the leaflets but above the papillary muscle heads. As the bioprosthesis is exposed and expands, the anchor tips are positioned under the annulus to capture the leaflets. Once sufficient TTVR positioning is achieved, the valve is fully deployed and released, with a careful system removal which avoids interaction with the valve [40].

### 5.2 Cardiovalve

The Cardiovalve (Cardiovalve Ltd) is an innovative three-leaflet bovine pericardium TTVR system which is designed for a 32-F transfemoral approach. It features a dual self-expanding nitinol frame with 24 grasping points for secure native valve anchoring. The atrial and ventricular frames are welded together, with the atrial frame has a Dacron fabric-covered flange for enhanced sealing and anchoring. The deployment involves three steps: exposing grasping legs in the RA, diving into the RV and grasping native leaflets, and exposing the atrial flange for full valve opening and posterior release [41].

### 5.3 LuX Valve

The LuX valve (Jenscare Biotechnology, Ningbo, China) represents a significant advancement in the field of TTVR. Operating on a 32 Fr flexible delivery system, this innovative device incorporates a bovine pericardial valve mounted on a nitinol stent. The stent itself features essential components, including an atrial disc, an interventricular septal anchor “tongue”, and two graspers covered with expanded polytetrafluoroethylene. Remarkably versatile, the LuX valve comes in four different sizes (from 30 to 55 mm) and offers eight disc sizes to accommodate annular diameters (from 25 to 50 mm). A distinctive feature of the LuX valve is its method of delivery, which can be executed through a femoral approach and a mini right thoracotomy, transjugular or transatrial approach.

Notably, this valve stands out as being radial force-independent, aiming to mitigate complications associated with force application during the procedure. This strategic design seeks to minimize risks related to conduction disturbances and RCA impingement. However, this advantage may be balanced by a potential trade-off, as there could be an increased risk of paravalvular regurgitation [42].

### 5.4 TriSol Valve 

The TriSol valve (TriSol Medical, Yokneam, Israel) is a self-expanding nitinol alloy frame with a bovine pericardial monoleaflet valve, designed for transcatheter deployment. In diastole, the valve opens, creating two large orifices for antegrade blood flow. In systole, ventricular contraction forces the leaflets to form a circular line of coaptation, resembling a mechanical double-disk valve, with a closing volume of approximately 5 mL. Six fixation arms anchor the valve to the native annulus, reducing the risk of impairment of the RCA flow. The conical shape and axial forces aim to prevent frame impingement on the conduction system. The device, crimped and loaded into the delivery system, is introduced via the transjugular approach, allowing collapsibility and repositioning until fixation arms fully expand. The prototype accommodates annulus dimensions of 40 to 53 mm, and while the presence of a CIED lead doesn’t prohibit the procedure, it may add complexity. There is only one case report [43] published about the valve implanted in a 71-year-old female with massive TR deemed high-risk for surgery, and experienced stable hemodynamics post-implantation, showcasing potential benefits for high-risk TR patients. Follow-up revealed sustained proper valve positioning, low transvalvular pressure gradients, and positive RV remodeling, suggesting a promising alternative to surgery.

### 5.5 Topaz 

The Topaz valve (TRiCares) represents an innovative approach to TTVR. It features a unique design consisting of a dual-stent frame crafted from nitinol. The outer stent serves to securely anchor the device within the native tricuspid apparatus while safeguarding the inner stent from potential deformation caused by RV contractions. Housing the valve itself, the inner stent, with its greater rigidity, is constructed to maintain the valve’s circular shape and integrity independently of the outer stent. Comprised of porcine pericardium, the three-leaflet valve functions autonomously from the outer stent. The system is delivered through a 29 Fr steerable introducer via the femoral vein, presently without the option of retrieval. The procedure involves positioning the introducer in the right atrium, aligning it with the tricuspid annulus, and then advancing the crimped valve to the RV apex for deployment in two stages—first the ventricular portion, followed by the atrial portion. Unlike traditional methods relying on radial force, the anchoring mechanism utilizes a series of anchors positioned below the annulus level, eliminating the need for valve oversizing. Currently, only one valve size is available, suitable for treating annulus diameters <45 mm as determined by diastolic CCT scans [44].

### 5.6 Intrepid

The Intrepid system (Medtronic, Minneapolis, MN, USA) is a 27 mm trileaflet bovine pericardial valve with a circular inner stent which can be deployed with 35 Fr delivery system through either transapical or transfemoral access. The delivery system is specifically designed for interventions involving both the MV and TV. Among its notable attributes are its capacity for steering in multiple directions and a unique deployment mechanism from the atrium to the ventricle.

Currently, there are two sizes available, with 42 mm and 48 mm valves undergoing clinical evaluation, while an extra-large valve is in the development phase [45].

### 5.7 VDyne 

The Vdyne valve (VDyneInc., Maple Grove, MN, USA), features a 30-mm porcine trifleaflet valve capable of accommodating tricuspid annuli with perimeters of up to 180 mm. The design includes securement features located strategically at the patient’s right ventricular outflow tract, ventricular free wall, and posterior septum, enabling slight oversizing. Instead of being radial, the valve is crimped or folded vertically. During implantation, the outer ventricular frame contracts by 25%, facilitating passage over the TA. Lateral guide deflection and steering help achieve a perpendicular position for easier placement. After delivery, the implant height along the frame’s lateral side is only 15 mm, and the system does not require pacing during deployment. Remarkably, the valve can be fully retrieved after expansion and positioning, allowing for a stability assessment before final decoupling [46].

## 6. Clinical Outcomes and Complications

### 6.1 Clinical Outcomes

Clinical outcomes from the available studies [40, 50] on TTVR showcases promising results, shedding light on the efficacy and safety of these interventions. In the TRISCEND study, focusing on Edwards EVOQUE TV replacement [41] 30-day results involving 56 patients revealed a remarkable 98% success rate in achieving mild or less TR.

In addition to reducing the severity of TR, patients significantly improved New York Heart Association (NYHA) functional class, 6-minute walk distance (6MWD), and Kansas City Cardiomyopathy Questionnaire (KCCQ) scores. The median time from implant insertion to release of EVOQUE valve was 70.1 minutes. Nonetheless, the composite major adverse event rate at 30 days was 26.8%, with contributing factors including one cardiovascular death due to a failed procedure, two reinterventions because of device embolization, one major vascular complication, and 15 cases of non-fatal severe bleeding. The rate of permanent PM implantation was 11.1%.

A high procedural success rate was achieved for the LUX device (45 out of 46 cases), with one instance of fatal RV perforation. The average procedure time was 150 minutes. Notably, at their six-month follow-up, 33 patients exhibited no or mild TR, indicating sustained positive outcomes [47].

Regarding the TriSol device, there is limited published data, with only one case report documenting its successful use. Further comprehensive studies are required to better understand its clinical outcomes [43].

### 6.2 Anticoagulation

There is currently no consensus on the optimal anticoagulation therapy or the duration of thrombosis prophylaxis. The TRISCEND II study protocol requires up to 6 months of anticoagulation therapy with warfarin, targeting an international normalized ratio (INR) of 2–3, along with a daily dose of 81 mg aspirin. Previous experience with an off-label balloon-expandable valve indicated that, out of 302 patients, 50% were discharged on oral anticoagulants, and the cumulative incidence of thrombosis was 0.033 (ranging from 0.015 to 0.061) at 3 years. An increased risk of thrombosis was associated with a higher post-TTVR gradient (heart rate 1.38 per mmHg). Due to the relatively high incidence of non-access site bleeding observed in the TRISCEND EFS study, careful patient selection to assess bleeding risk is crucial [40].

### 6.3 Complications 

In the TTVR field, the comprehensive spectrum of potential complications remains shrouded in uncertainty. However, it is pertinent to acknowledge that, akin to other transcatheter interventions, all TTVR devices carry inherent risks and necessitate due precautions that are worth noting.

#### 6.3.1 Valve-Related Complications

Improper anchoring, a critical procedural aspect, holds the potential to instigate a cascade of complications, ranging from device malfunction to paravalvular leak, valve embolism or thrombosis. The intricacies of TV anatomy demand meticulous attention to anchoring details to mitigate these risks effectively. Notably, the unique hemodynamic environment of the right heart chambers, characterized by reduced blood flow speed compared to the left side, introduces an additional layer of complexity. This disparity in blood flow dynamics is believed to elevate the risk of valvular thrombus formation, emphasizing the importance of precision and vigilance in addressing anchoring considerations during TTVR procedures [48].

#### 6.3.2 Bleedings 

Bleeding complications following transcatheter TTVR are a critical aspect of post-procedural care. The initiation of anticoagulation post-TTVR is highly recommended, with the timing carefully balanced to ensure safety while considering bleeding risks associated with the patient’s medical history, access-site considerations, and potential gastrointestinal (GI) bleeding related to TEE [49]. Often, anticoagulation is already warranted in this patient cohort, frequently due to concurrent indications such as atrial fibrillation. Despite the implementation of anticoagulation strategies, all-cause major bleeding remains a common occurrence, afflicting approximately 10%–15% of patients across various platforms.

GI injury, mainly represented by bleeding, is recognized as a significant risk factor during procedures, especially when there are extended procedure times and poor imaging quality. Although vascular injury and bleeding at the access site are extremely rare, their potential occurrence highlights the importance of careful monitoring throughout the TTVR procedure [48].

In the realm of preventive measures, the consideration of additional antiplatelet therapy is pivotal for averting complications such as leaflet thrombosis. The comprehensive management of bleeding risks post-TTVR demands a nuanced approach, emphasizing the delicate balance between anticoagulation benefits and potential adverse events, with continuous evaluation and refinement of strategies to enhance patient outcomes in this evolving landscape of TV interventions.

#### 6.3.3 Conduction System Disturbances 

As previously outlined, positioning a TTVR device could lead to conduction system injury with a potential need for permanent PM and, moreover, this will exclude patients from conventional ventricular lead placement. Implantation of a leadless PM or coronary sinus lead may be a valuable alternative to overcome this issue but this must be carefully planned. Ventricular lead placement is common in severe TR population [47]. In fact, in the TRISCEND EFS study, 34% of participants had prior CIED leads before the procedure. Moreover, THV implantation may compromise ventricular leads in patients which are PM dependent or who had prior implanted cardioverter defibrillator (ICD) implantation for secondary prevention of sudden heart death (SHD) [50].

### 6.4 Right Heart Failure 

The RV, due to its anatomic and physiological features, is well-adapted to handle volume overload conditions (such as severe TR) but does not tolerate pressure overload, especially if this is acute. Chronic severe TR may cause RV dilation and could hide an underlying systolic dysfunction.

Therefore, an immediate correction of severe TR can potentially unmask underlying RV dysfunction and may result in hemodynamic instability. This occurs because eliminating TR suddenly increases afterload on the RV, which may exacerbate RV failure or at the very least prevent improvement. This phenomenon is more probable in cases of functional TR, especially when associated with severe pulmonary hypertension. The overall risk of afterload mismatch in percutaneous interventions is generally lower compared to open-heart surgery [47].

In the TRISCEND trial, early right heart failure occurred in 2 patients, which required inotropic support in the post-implantation phase [40].

RV failure after correction of severe TR is a serious and potentially fatal complication which needs to be adequately prevented with careful patient selection and also involvement of heart failure experts, especially for high risk patients (e.g., those with preexisting systolic dysfunction and massive or torrential TR) [47].

Further studies are required to identify high-risk patients who may develop right heart failure following TTVR and to develop strategies for preventing and managing this complication.

## 7. Future Direction and Ongoing Trials 

The field of TTVR is swiftly advancing to address the substantial unmet need resulting from the undertreatment of TR. Progress in disease understanding and assessment tools enables the standardization of TR definitions, severity criteria, and clinical endpoints. The introduction of a novel five-grade TR severity scale enhances procedural clarity and facilitates the evaluation of TR reduction’s impact on clinical outcomes. As TTVR advances, ongoing studies explore questions related to device selection, durability, and comparative safety (Table 2). Despite challenges such as prolonged procedural times and anatomical complexity, innovations in imaging techniques like intracardiac echocardiography (ICE) and continuous device enhancements are anticipated to improve safety and usability in TTVR. The evolving landscape aims to replicate surgical outcomes and minimize unnecessary procedures, particularly in the context of multi-valvular diseases. Additionally, numerous ongoing trials contribute to the continuous development of this specific field, although patient enrollment in these clinical trials is strongly limited by patients’ ineligibility for the procedure due to anatomical and clinical characteristics (such as chronic kidney disease):

**Table 2.** **Current Trials on Transcatheter Tricuspid Valve Replacement (TTVR)**.

Ongoing trials	Interventional group	Primary endpoint	Estimated enrollment	Actual enrollment	Completion date
Travel II (NCT05194423)	Subjects who received TTVR with LuX-Valve and delivery system via jugular vein.	Death, TR measured with echocardiography in core lab reduces at least 2 grades.	150 patients	96 patients	March 2027
TRISCEND II (NCT04482062)	Treatment with the Edwards EVOQUE Tricuspid TTVR System.	Freedom from device or procedure-related adverse events.	200 patients	136 patients	December 2025
Triplace (NCT NCT06033274)	Patients undergoing orthotopic TTVR for clinically significant TV disease.	Freedom from device-related complications; Reduction in TR Severity.	300 patients	/	August 2028
TARGET (cardiovalve)	Symptomatic subjects (NYHA Class ≥II–IVa) with severe TR requiring valve replacement or repair who are at high risk for open chest surgery undergoing Cardiovalve TR replacement.	Safety and Performance of the Cardiovalve TR replacement system for TR.	150 patients	22 patients	January 2028
TRiCares Topaz Transfemoral Tricuspid Heart Valve Replacement System First In Human Trial (TRICURE)	Insights into the safety profile and performance of the Topaz TTVR system intended for transfemoral access in patients with severe TR symptomatic (NYHA ≥II) not eligible for surgery.	Hierarchical composite endpoint including all-cause mortality, re-hospitalization for heart failure, re-intervention for failed tricuspid intervention, and KCCQ worsening at 30 days.	20 patients	/	May 2029
Clinical Safety and Efficacy of the VDyne Transcatheter Tricuspid Valve Replacement System for the Treatment of Tricuspid Regurgitation (VISTA-US)	Patients undergoing orthotopic TTVR for clinically significant tricuspid valve disease with V-Dyne valve.	30-days procedure-related MAEs; changes in TR; changes in NYHA class; changes in functional capacity (6MWD); changes in quality of life (KCCQ score).	30 patients	/	November 2025

Legend: TR, tricuspid regurgitation; NYHA, New York Heart Association; KCCQ, Kansas City Cardiomyopathy Questionnaire; MAEs, major adverse events; 6MWD, 6-minutes walking distance; TV, tricuspid valve.

### 7.1 The TRAVEL TRIAL: Transcatheter Right Atrial-ventricular Valve Replacement with LuX-Valve (NCT04436653)

The TRAVEL trial is a prospective, multi-center, single-arm study which aims to assess the safety and effectiveness of the LuX-Valve TTVR and delivery system in symptomatic patients with severe TR and high surgical risk. The minimum size required to complete enrollment is 150 subjects and a follow up of five years. The primary endpoints include death from any cause and a reduction in TR (by at least 2 points measured with echocardiography in a core lab). Secondary endpoints encompass device or procedure-related adverse events, major adverse events, changes in NYHA classification, and alterations in Quality of Life assessed through the 6MWD. The study completion is estimated for June 2026. There is also the Travel II trial (NCT05194423) which has the same design but is evaluating the LuX-Valve implantation via the jugular vein, the completion of which is estimated for March 2027.

### 7.2 Triscend II Pivotal Trial (NCT04482062)

This prospective, multi-center, randomized controlled pivotal clinical trial is designed to assess the safety and efficacy of the EVOQUE System when used alongside optimal medical therapy (OMT), compared to OMT alone, in patients with severe or greater TR. Follow-up assessments will occur at discharge, 30 days, 3 months, 6 months, and annually for up to 5 years. The primary endpoints include reducing the TR grade and a composite endpoint that covers improvements in the KCCQ, NYHA functional class, and 6MWD. Secondary endpoints consist of the major adverse events (MAE) rate and another composite endpoint that includes all-cause mortality, RV assist device (RVAD) implantation or heart transplant, TV interventions, heart failure hospitalizations, and enhancements in KCCQ, NYHA functional class, and 6MWD. The study is projected to be completed by December 2029.

### 7.3 VISTA-US Clinical Trial (NCT05848284)

The VISTA-US study (Clinical Safety and Efficacy of the VDyne Transcatheter Tricuspid Valve Replacement System for the Treatment of Tricuspid Regurgitation) is an open label, single arm clinical trial which focuses on the VDyne TTVR, comprising a bioprosthetic implantable TV and associated delivery and retrieval systems. The required sample size is of 30 patients and the enrollment is estimated to be completed by November 2025. The primary outcomes assessed within 30 days post-procedure include the percentage of subjects experiencing Device- and/or Procedure-related MAE, changes in TV regurgitation from baseline, alterations in symptom status (NYHA class), shifts in functional capacity (6MWD), and variations in quality of life (KCCQ score). Secondary outcomes, evaluated from 30 days to 1 year post-procedure, include the continuation of MAE assessment, changes in TR, alterations in RV measurements, HF hospitalization rates, shifts in symptom status and functional capacity, and changes in quality of life scores.

## 8. Limitations

The present review has some limitations which have to be elucidated. First of all, there is limited long-term research for TTVR devices. In fact, most available studies are based on short-term data, with few long-term results regarding durability of the prosthesis, long-term complications and clinical outcomes. Moreover, there is a lack of randomized controlled trials in this setting, with many studies with an observational or retrospective design, leading to potential biases. Finally, there is continuous innovation and evolution in TTVR devices and implantation techniques, therefore the older studies taken into account may not reflect the latest technological innovations.

## 9. Conclusions

TR is a potentially lethal condition and represents a significant clinical challenge both for clinical and interventional cardiologists. Transcatheter interventions are currently a valid alternative to surgery, especially in patients with prohibitive or high surgical risk. Among those therapies, TTVR is an emergent procedure which can be an option, especially in patients not eligible for surgery or TEER. Multimodality imaging is crucial in evaluating procedure feasibility, in particular assessing TV anatomy, TR mechanism and severity as well as the size and function of the right chambers. The main issues related to TTVR are represented by the lack of long-term data regarding the durability of the valve, the risk of thrombosis and the optimal strategy of antithrombotic therapy. Moreover, TTVR carries the risk of potential complications such as high-degree heart block requiring permanent PM implantation, bleeding and acute right heart failure due to afterload mismatch.

Currently, several randomized controlled trials are ongoing to evaluate the efficacy, safety and durability of multiple TTVR devices in managing severe TR and, hopefully, they will clarify the uncertainties in this field.

## Abbreviations

6MWD, 6-minute walking distance; AF, atrial fibrillation; CCT, cardiac computed tomography; CIED, cardiac implantable electronic devices; CMR, cardiac magnetic resonance; Ea, arterial elastance; Ees, end systolic elastance; EROA, effective regurgitant orifice area; FAC, fractional area change; GI, gastrointestinal; HF, heart failure; HFpEF, heart failure with preserved ejection fraction; HFrEF, heart failure with reduced ejection fraction; ICD, implanted cardioverter defibrillator; ICE, intracardiac echography; IVC, inferior vena cava; KCCQ, Kansas City Cardiomyopathy Questionnaire; LV, left ventricle; MAE, major adverse event; MR, mitral regurgitation; MV, mitral valve; NYHA, New York Heart Association; OMT, optimal medical therapy; PA, pulmonary artery; PASP, pulmonary artery systolic pressure; PISA, proximal isovelocity surface area; PM, pacemaker; RA, right atrium; RCA, right coronary artery; RHF, right heart failure; RV, right ventricle; RVAD, right ventricular assist device; SCD, sudden cardiac death; SGLT2-i, sodium–glucose cotransporter 2 inhibitors; SVC, superior vena cava; TA, tricuspid annulus; TAPSE, tricuspid annulus plane systolic excursion; TEER, transcatheter edge-to-edge repair; TEE, transesophageal echocardiography; TR, tricuspid regurgitation; TTE, transthoracic echocardiography; TTVI, transcatheter tricuspid valve intervention; TTVR, transcatheter tricuspid valve replacement; TV, tricuspid valve; VC, vena contracta.

## References

[1] Topilsky Y, Maltais S, Medina Inojosa J, Oguz D, Michelena H, Maalouf J (2019). Burden of Tricuspid Regurgitation in Patients Diagnosed in the Community Setting. *JACC. Cardiovascular Imaging*.

[2] Mutlak D, Lessick J, Reisner SA, Aronson D, Dabbah S, Agmon Y (2007). Echocardiography-based spectrum of severe tricuspid regurgitation: the frequency of apparently idiopathic tricuspid regurgitation. *Journal of the American Society of Echocardiography: Official Publication of the American Society of Echocardiography*.

[3] Nath J, Foster E, Heidenreich PA (2004). Impact of tricuspid regurgitation on long-term survival. *Journal of the American College of Cardiology*.

[4] Koelling TM, Aaronson KD, Cody RJ, Bach DS, Armstrong WF (2002). Prognostic significance of mitral regurgitation and tricuspid regurgitation in patients with left ventricular systolic dysfunction. *American Heart Journal*.

[5] Cohen SR, Sell JE, McIntosh CL, Clark RE (1987). Tricuspid regurgitation in patients with acquired, chronic, pure mitral regurgitation. II. Nonoperative management, tricuspid valve annuloplasty, and tricuspid valve replacement. *The Journal of Thoracic and Cardiovascular Surgery*.

[6] Chorin E, Rozenbaum Z, Topilsky Y, Konigstein M, Ziv-Baran T, Richert E (2020). Tricuspid regurgitation and long-term clinical outcomes. *European Heart Journal. Cardiovascular Imaging*.

[7] Hahn RT, Badano LP, Bartko PE, Muraru D, Maisano F, Zamorano JL (2022). Tricuspid regurgitation: recent advances in understanding pathophysiology, severity grading and outcome. *European Heart Journal. Cardiovascular Imaging*.

[8] Taramasso M, Benfari G, Van Der Bijl P, Alessandrini H, Attinger-Toller A, Biasco L (2019). Transcatheter Versus Medical Treatment of Patients with Symptomatic Severe Tricuspid Regurgitation. *Journal of the American College of Cardiology*.

[9] Nishimura RA, Otto CM, Bonow RO, Carabello BA, Erwin JP, Fleisher LA (2017). 2017 AHA/ACC Focused Update of the 2014 AHA/ACC Guideline for the Management of Patients with Valvular Heart Disease: A Report of the American College of Cardiology/American Heart Association Task Force on Clinical Practice Guidelines. *Circulation*.

[10] Taramasso M, Pozzoli A, Basso C, Thiene G, Denti P, Kuwata S (2018). Compare and contrast tricuspid and mitral valve anatomy: interventional perspectives for transcatheter tricuspid valve therapies. *EuroIntervention: Journal of EuroPCR in Collaboration with the Working Group on Interventional Cardiology of the European Society of Cardiology*.

[11] Asmarats L, Puri R, Latib A, Navia JL, Rodés-Cabau J (2018). Transcatheter Tricuspid Valve Interventions: Landscape, Challenges, and Future Directions. *Journal of the American College of Cardiology*.

[12] Besler C, Orban M, Rommel KP, Braun D, Patel M, Hagl C (2018). Predictors of Procedural and Clinical Outcomes in Patients with Symptomatic Tricuspid Regurgitation Undergoing Transcatheter Edge-to-Edge Repair. *JACC. Cardiovascular Interventions*.

[13] Chang JD, Manning WJ, Ebrille E, Zimetbaum PJ (2017). Tricuspid Valve Dysfunction Following Pacemaker or Cardioverter-Defibrillator Implantation. *Journal of the American College of Cardiology*.

[14] Prihadi EA, Delgado V, Hahn RT, Leipsic J, Min JK, Bax JJ (2018). Imaging Needs in Novel Transcatheter Tricuspid Valve Interventions. *JACC. Cardiovascular Imaging*.

[15] Badano LP, Caravita S, Rella V, Guida V, Parati G, Muraru D (2021). The Added Value of 3-Dimensional Echocardiography to Understand the Pathophysiology of Functional Tricuspid Regurgitation. *JACC. Cardiovascular Imaging*.

[16] Muraru D, Hahn RT, Soliman OI, Faletra FF, Basso C, Badano LP (2019). 3-Dimensional Echocardiography in Imaging the Tricuspid Valve. *JACC. Cardiovascular Imaging*.

[17] Hahn RT, Zamorano JL (2017). The need for a new tricuspid regurgitation grading scheme. *European Heart Journal. Cardiovascular Imaging*.

[18] Dahou A, Ong G, Hamid N, Avenatti E, Yao J, Hahn RT (2019). Quantifying Tricuspid Regurgitation Severity: A Comparison of Proximal Isovelocity Surface Area and Novel Quantitative Doppler Methods. *JACC. Cardiovascular Imaging*.

[19] Utsunomiya H, Harada Y, Susawa H, Ueda Y, Izumi K, Itakura K (2020). Tricuspid valve geometry and right heart remodelling: insights into the mechanism of atrial functional tricuspid regurgitation. *European Heart Journal. Cardiovascular Imaging*.

[20] Praz F, Khalique OK, Dos Reis Macedo LG, Pulerwitz TC, Jantz J, Wu IY (2018). Comparison between Three-Dimensional Echocardiography and Computed Tomography for Comprehensive Tricuspid Annulus and Valve Assessment in Severe Tricuspid Regurgitation: Implications for Tricuspid Regurgitation Grading and Transcatheter Therapies. *Journal of the American Society of Echocardiography: Official Publication of the American Society of Echocardiography*.

[21] Lang RM, Badano LP, Victor MA, Afilalo J, Armstrong A, Ernande L (2015). Recommendations for cardiac chamber quantification by echocardiography in adults: An update from the American Society of Echocardiography and the European Association of Cardiovascular Imaging. *Journal of the American Society of Echocardiography*.

[22] Nemoto N, Lesser JR, Pedersen WR, Sorajja P, Spinner E, Garberich RF (2015). Pathogenic structural heart changes in early tricuspid regurgitation. *The Journal of Thoracic and Cardiovascular Surgery*.

[23] Hahn RT, Weckbach LT, Noack T, Hamid N, Kitamura M, Bae R (2021). Proposal for a Standard Echocardiographic Tricuspid Valve Nomenclature. *JACC. Cardiovascular Imaging*.

[24] O’Neill B, Wang DD, Pantelic M, Song T, Guerrero M, Greenbaum A (2015). Transcatheter Caval Valve Implantation Using Multimodality Imaging. *JACC. Cardiovascular Imaging*.

[25] Blusztein DI, Hahn RT (2023). New therapeutic approach for tricuspid regurgitation: Transcatheter tricuspid valve replacement or repair. *Frontiers in Cardiovascular Medicine*.

[26] Naeije R, Tello K, D’Alto M (2023). Tricuspid Regurgitation: Right Ventricular Volume Versus Pressure Load. *Current Heart Failure Reports*.

[27] Schmeisser A, Rauwolf T, Groscheck T, Kropf S, Luani B, Tanev I (2021). Pressure-volume loop validation of TAPSE/PASP for right ventricular arterial coupling in heart failure with pulmonary hypertension. *European Heart Journal. Cardiovascular Imaging*.

[28] Brener MI, Lurz P, Hausleiter J, Rodés-Cabau J, Fam N, Kodali SK (2022). Right Ventricular-Pulmonary Arterial Coupling and Afterload Reserve in Patients Undergoing Transcatheter Tricuspid Valve Repair. *Journal of the American College of Cardiology*.

[29] Lurz P, Orban M, Besler C, Braun D, Schlotter F, Noack T (2020). Clinical characteristics, diagnosis, and risk stratification of pulmonary hypertension in severe tricuspid regurgitation and implications for transcatheter tricuspid valve repair. *European Heart Journal*.

[30] Sugiura A, Tanaka T, Kavsur R, Öztürk C, Silaschi M, Goto T (2024). Refining accuracy of RV-PA coupling in patients undergoing transcatheter tricuspid valve treatment. *Clinical Research in Cardiology: Official Journal of the German Cardiac Society*.

[31] Min SY, Song JM, Kim JH, Jang MK, Kim YJ, Song H (2010). Geometric changes after tricuspid annuloplasty and predictors of residual tricuspid regurgitation: a real-time three-dimensional echocardiography study. *European Heart Journal*.

[32] van Rosendael PJ, Kamperidis V, Kong WKF, van Rosendael AR, van der Kley F, Ajmone Marsan N (2017). Computed tomography for planning transcatheter tricuspid valve therapy. *European Heart Journal*.

[33] Agricola E, Asmarats L, Maisano F, Cavalcante JL, Liu S, Milla F (2021). Imaging for Tricuspid Valve Repair and Replacement. *JACC. Cardiovascular Imaging*.

[34] Takx RAP, Moscariello A, Schoepf UJ, Barraza JM, Nance JW, Bastarrika G (2012). Quantification of left and right ventricular function and myocardial mass: comparison of low-radiation dose 2nd generation dual-source CT and cardiac MRI. *European Journal of Radiology*.

[35] McDonagh TA, Metra M, Adamo M, Gardner RS, Baumbach A, Böhm M (2022). 2021 ESC Guidelines for the diagnosis and treatment of acute and chronic heart failure: Developed by the Task Force for the diagnosis and treatment of acute and chronic heart failure of the European Society of Cardiology (ESC). With the special contribution of the Heart Failure Association (HFA) of the ESC. *European Journal of Heart Failure*.

[36] Metra M, Tomasoni D, Adamo M, Bayes-Genis A, Filippatos G, Abdelhamid M (2023). Worsening of chronic heart failure: definition, epidemiology, management and prevention. A clinical consensus statement by the Heart Failure Association of the European Society of Cardiology. *European Journal of Heart Failure*.

[37] Correale M, Mallardi A, Mazzeo P, Tricarico L, Diella C, Romano V (2020). Sacubitril/valsartan improves right ventricular function in a real-life population of patients with chronic heart failure: The Daunia Heart Failure Registry. *International Journal of Cardiology. Heart & Vasculature*.

[38] Boehm M, Arnold N, Braithwaite A, Pickworth J, Lu C, Novoyatleva T (2018). Eplerenone attenuates pathological pulmonary vascular rather than right ventricular remodeling in pulmonary arterial hypertension. *BMC Pulmonary Medicine*.

[39] Mustapic I, Bakovic D, Susilovic Grabovac Z, Borovac JA (2022). Impact of SGLT2 Inhibitor Therapy on Right Ventricular Function in Patients with Heart Failure and Reduced Ejection Fraction. *Journal of Clinical Medicine*.

[40] Kodali S, Hahn RT, George I, Davidson CJ, Narang A, Zahr F (2022). Transfemoral Tricuspid Valve Replacement in Patients with Tricuspid Regurgitation: TRISCEND Study 30-Day Results. *JACC. Cardiovascular Interventions*.

[41] Caneiro-Queija B, Estévez-Loureiro R, Piñón-Esteban M, Barreiro-Pérez M, Baz-Alonso JA, Íñiguez-Romo A (2023). Transfemoral transcatheter tricuspid valve replacement with the Cardiovalve system. *Revista Espanola De Cardiologia (English Ed.)*.

[42] Wei W, Ning L, Xiaoping N, Zhiyun X, Bailing L, Chengliang C (2022). Hemodynamics of transcatheter tricuspid valve replacement with Lux-Valve. *Frontiers in Cardiovascular Medicine*.

[43] Vaturi M, Vaknin-Assa H, Shapira Y, Perl L, Levi A, Koren A (2021). First-in-Human Percutaneous Transcatheter Tricuspid Valve Replacement with a Novel Valve. *JACC. Case Reports*.

[44] Teiger E, Nejjari M, Lim P, Ruf T, Blanke P, Schäfer U (2022). First-in-human implantation of the Topaz transcatheter tricuspid valve replacement system. *EuroIntervention: Journal of EuroPCR in Collaboration with the Working Group on Interventional Cardiology of the European Society of Cardiology*.

[45] Blusztein DI, Hahn RT, Godoy Rivas C, George I, Kodali SK (2023). Transcatheter Tricuspid Valve Replacement with Novel Self-Expanding Valve: Secure Fixation in Insecure Anatomy. *JACC. Case Reports*.

[46] Sorajja P, Gorgorishvili I, Burns M, Buysschaert I, Debonnaire P, Van der Heyden J (2023). First-in-human description of a novel transcatheter tricuspid valve prosthesis to preserve the asymmetric shape of the right ventricle. *EuroIntervention: Journal of EuroPCR in Collaboration with the Working Group on Interventional Cardiology of the European Society of Cardiology*.

[47] Lu FL, An Z, Ma Y, Song ZG, Cai CL, Li BL (2021). Transcatheter tricuspid valve replacement in patients with severe tricuspid regurgitation. *Heart (British Cardiac Society)*.

[48] Goldberg YH, Ho E, Chau M, Latib A (2021). Update on Transcatheter Tricuspid Valve Replacement Therapies. *Frontiers in Cardiovascular Medicine*.

[49] Freitas-Ferraz AB, Bernier M, Vaillancourt R, Ugalde PA, Nicodème F, Paradis JM (2020). Safety of Transesophageal Echocardiography to Guide Structural Cardiac Interventions. *Journal of the American College of Cardiology*.

[50] Kodali S, Hahn RT, Makkar R, Makar M, Davidson CJ, Puthumana JJ (2023). Transfemoral tricuspid valve replacement and one-year outcomes: the TRISCEND study. *European Heart Journal*.

